# Supplementary material for: Use of Bladder-Related Medication in Non-Muscle Invasive Bladder Cancer Patients
Source: Cancers (Basel). 2024 May 20;16(10):1936. doi: 10.3390/cancers16101936 (PMC11119777; doi:10.3390/cancers16101936)
Supplement: Supplementary file 1 [file cancers-16-01936-s001.zip › cancers-2944188-supplementary.pdf]

Supplementary Table S1. Codebook.

Registry	*Description*	*Code*
The Danish National Patient Registry (DNPR)	**Diagnose codes:**	**The International Classification of Diseases, 10th Edition (ICD-10)**
	Cancer of the urinary bladder	DC67*
	Carcinoma in situ in the urinary bladder	DD090
	Non-invasive papillary tumor (Ta) in the urinary bladder	DD095
	Neoplasm of low malignant potential (PUNLMP)	DD303
	**Other allowed cancer diagnoses:**	
	Prostate cancer	DC619
	Non-melanoma skin cancer	DC44*
	**Procedure codes:**	
	Cystoscopy	KUKC02
	Cystoscopy with biopsy	KUKC05
	Transurethral resection of the bladder (TURB)	KKCD32*
	Bacillus Calmette-Guerin vaccine (BCG) instillation in the urinary bladder	BJHE11
	Instillation of cytostatic in the urinary bladder	BJHE12
	Instillation of mitomycin C (MMC) in the urinary bladder	BJHE12A
	Cystectomy	KKCC*
Danish Pathology Registry (DPR)	**Pathological description of neoplastic lesions of the urinary bladder**	**SNOMED codes**
	Urinary bladder	T74*
	Urothelial carcinoma in situ	M81202
	Urothelial carcinoma	M81203
	PUNLMP	M81301
	**Grade:**	
	Low grade (Corresponds Bergkvist classification ÆYYY11 and ÆYYY12)	ÆYY111
	High grade (Corresponds Bergkvist classification ÆYYY13 and ÆYYY14)	ÆYY113
	**pTNM stage:**	
	pTis	ÆF1810
	pTa	ÆF181a
	pT1	ÆF1830
	pT1a (Danish sub-classification of pT1)	ÆF1831
	pT1b (Danish sub-classification of pT1)	ÆF1832
	pT2	ÆF1840
	pT2a	ÆF1841
	pT2b	ÆF1842
	pT3	ÆF1850
	pT4a	ÆF1861
	pT4b	ÆF1862
The National Prescription Registry (NPR)	**Medicine used in treating frequency and urinary incontinence**	**Anatomical Therapeutic Chemical (ATC)**
	Anticholinergics and β3-agonists	G04BD*
	**Antibiotics used in the treatment of urinary tract infection:**	**ATC**
	Pivmecillinam	J01CA08
	Trimethoprim	J01EA01
	Sulfamethiziole	J01EB02
	Nitrofurantoin	J01XE01
	Ciprofloxacin	J01MA02
	Bioclavid	J01CR02

Cause of death register (DAR)	***Direct cause of death*** **or** ***Contributing cause of death***	**ACME (Automated Classification of Medical Entities) via ICD-10**
	Bladder cancer	B-027

Supplementary Tables S2–S4: Baseline characteristics distributed on exposure

Supplementary Table S2. Exposure: TURB load within the first 5 years from NMIBC-diagnosis.

**Event of interest: Bladder relaxing agents**	**Group 1 (1 TURB)**	**Group 2 (2 – 4 TURBs)**	**Group 3 (≥ 5 TURBs)**
**Registrations within exposure groups (N)**	17,774	9159	1675
**Sex:**			
Female	24.2%	25.1%	29.7%
Male	75.8%	74.9%	70.3%
**Baseline age, median (IQR):**	70 (63,77)	70 (63,76)	69 (62,75)
**Primary histology, freq. (%)**			
PUNLMP	886 (4.98%)	470 (5.13%)	129 (7.70%)
pTa LG	8032 (45.19%)	3799 (41.48%)	825 (49.25%)
pTa (grade unknown)	785 (4.42%)	378 (4.13%)	94 (5.61%)
pTa HG	2470 (13.90%)	1327 (14.49%)	220 (13.13%)
CIS	1085 (6.10%)	534 (5.83%)	71 (4.24%)
pTa, concomitant CIS	570 (3.21%)	296 (3.23%)	34 (2.03%)
pT1a	1485 (8.35%)	901 (9.84%)	126 (7.52%)
pT1 (sub-division unknown)	1491 (8.39%)	925 (10.10%)	145 (8.66%)
pT1b	970 (5.46%)	529 (5.78%)	31 (1.85%)
**Any collection of prescriptions before NMIBC-diagnosis, freq. (%)**	1203 (6.77%)	533 (5.82%)	67 (4.00%)

**Event of interest: Cystitis-relevant antibiotics**			
**Registrations within exposure groups (N)**	17,774	7507	946
**Sex:**			
Female	24.2%	24.7%	27.6%
Male	75.8%	75.3%	72.4%
**Baseline age, median (IQR):**	70 (63, 77)	69 (62,76)	68 (61,75)
**Primary histology, freq. (%)**			
PUNLMP	886 (4.98%)	401 (5.34%)	87 (9.20%)
pTa LG	8032 (45.19%)	3122 (41.59%)	487 (51.48%)
pTa (grade unknown)	785 (4.42%)	323 (4.30%)	59 (6.24%)
pTa HG	2470 (13.90%)	1042 (13.88%)	115 (12.16%)
CIS	1085 (6.10%)	419 (5.58%)	33 (3.49%)
pTa, concomitant CIS	570 (3.21%)	209 (2.78%)	13 (1.37%)
pT1a	1485 (8.35%)	751 (10.00%)	56 (5.92%)
pT1 (sub-division unknown)	1491 (8.39%)	784 (10.44%)	79 (8.35%)
pT1b	970 (5.46%)	456 (6.07%)	17 (1.80%)
**Any collection of prescriptions before NMIBC diagnosis, freq. (%)**	5720 (56.25%)	2089 (49.03%)	208 (40.47%)

Supplementary Table S3. Exposure: BCG instillations within the first five years from NMIBC diagnosis.

**Event of interest: Bladder relaxing agents**	**No BCG installation**	**BCG installations**
**Registrations within exposure groups (N)**	17,751	2,995
**Sex:**		
Female	24.2%	21.4%
Male	75.8%	78.6%
**Baseline age, median (IQR):**	70 (63,77)	69 (63,75)
**Primary histology, freq. (%)**		
PUNLMP	886 (4.99%)	61 (2.04%)
pTa LG	8024 (45.20%)	530 (17.70%)
pTa (grade unknown)	785 (4.42%)	44 (1.47%)
pTa HG	2468 (13.90%)	540 (18.03%)
CIS	1074 (6.05%)	378 (12.62%)
pTa, concomitant CIS	570 (3.21%)	317 (10.58%)
pT1a	1485 (8.37%)	555 (18.53%)
pT1 (sub-division unknown)	1489 (8.39%)	383 (12.79%)
pT1b	970 (5.46%)	187 (6.24%)
**Any collection of prescriptions before NMIBC diagnosis, freq. (%)**	1197 (6.74%)	224 (7.48%)
**Event of interest: Cystitis-relevant antibiotics**		
**Registrations within exposure groups (N)**	17,751	2166
**Sex:**		
Female	24.2%	21.1%
Male	75.8%	78.9%
**Baseline age, median (IQR):**	70 (63,77)	69 (63,75)
**Primary histology, freq. (%)**		
PUNLMP	886 (4.99%)	36 (1.66%)
pTa LG	8024 (45.20%)	358 (16.53%)
pTa (grade unknown)	785 (4.42%)	31 (1.43%)
pTa HG	2468 (13.90%)	387 (17.87%)
CIS	1074 (6.05%)	308 (14.22%)
pTa, concomitant CIS	570 (3.21%)	244 (11.27 %)
pT1a	1485 (8.37%)	395 (18.24%)
pT1 (sub-division unknown)	1489 (8.39%)	267 (12.33%)
pT1b	970 (5.46%)	140 (6.46%)
**Any collection of prescriptions before NMIBC diagnosis, freq. (%)**	5708 (56.23%)	642 (54.09%)

Supplementary Table S4. Exposure: MMC instillations within the first five years from NMIBC diagnosis.

Event of interest: Bladder relaxing agents	No MMC installation	MMC installations
**Registrations within exposure groups (N)**	17,756	429
**Sex:**		
Female	24.25%	27.3%
Male	75.75%	72.7%
**Age median,(IQR) til tiden t0:**	70 (63,77)	68 (62,76)
**Primary histology, freq. (%)**		
PUNLMP	885 (4.98%)	9 (2.10%)
pTa LG	8024 (45.19%)	226 (52.68%)
pTa (grade unknown)	784 (4.42%)	20 (4.66%)
pTa HG	2469 (13.91%)	69 (16.08%)
CIS	1083 (6.10%)	33 (7.69%)
pTa, concomitant CIS	568 (3.20%)	15 (3.50%)
pT1a	1485 (8.36%)	25 (5.83%)
pT1 (sub-division unknown)	1489 (8.39%)	24 (5.59%)
pT1b	969 (5.46%)	8 (1.86%)
**Any collection of prescriptions before NMIBC diagnosis, freq. (%)**	1200 (6.76%)	27 (6.29%)
**Event of interest: Cystitis-relevant antibiotics**		
**Registrations within exposure groups (N)**	17,756	304
**Sex:**		
**Female**	24.25%	25.33%
**Male**	75.75%	74.67%
**Age median,(IQR) til tiden t0:**	70 (63, 77)	68 (61.5, 75)
**Primary histology, freq. (%)**		
PUNLMP	885 (4.98%)	< 5 (1.32%)
pTa LG	8024 (45.19%)	171 (56.25%)
pTa (grade unknown)	784 (4.42%)	14 (4.61%)
pTa HG	2469 (13.91%)	48 (15.79%)
CIS	1083 (6.10%)	17 (5.59%)
pTa, concomitant CIS	568 (3.20%)	10 (3.29%)
pT1a	1485 (8.36%)	18 (5.92%)
pT1 (sub-division unknown)	1489 (8.39%)	17 (5.59%)
pT1b	969 (5.46%)	5 (1.64%)
**Any collection of prescriptions before NMIBC diagnosis, freq. (%)**	5709 (56.22%)	92 (52.27%)
